# Substance Use is Associated With College Students' Acute Parasympathetic Nervous System Responses to Challenge

**DOI:** 10.1002/smi.70002

**Published:** 2025-01-20

**Authors:** Danny Rahal, Violet F. Kwan, Kristin J. Perry

**Affiliations:** ^1^ University of California Santa Cruz Santa Cruz California USA; ^2^ University of California Los Angeles Los Angeles California USA; ^3^ Prevention Science Institute University of Oregon Eugene Oregon USA

**Keywords:** addiction, autonomic nervous system, parasympathetic nervous system, physiological response, substance abuse

## Abstract

College students use substances for varied reasons, including to cope with stress. The parasympathetic nervous system (PNS) regulates bodily functions to promote energy conservation (the ‘rest and digest’ response), and individuals differ in their physiological sensitivity to challenge. It remains unclear whether greater PNS responses (i.e., declines in PNS activity, termed vagal withdrawal) to challenge could suggest difficulty regulating and thereby confer risk for using substances in community samples. We hypothesised that lower resting PNS activity and greater PNS responses to a challenge task would be associated with more frequent substance use (i.e., alcohol use, binge drinking, cannabis use). College students (*N* = 152; *M*age = 20.5, *SD* = 3.2; 73.8% female) reported their past month frequency of substance use and completed a laboratory‐based challenge task while having an electrocardiogram administered to derive respiratory sinus arrhythmia (RSA), a measure of PNS activity. They watched a 4‐min neutral video (resting baseline) and then traced a star with their nondominant hand while only seeing the mirror reflection of their hand (challenge). Higher resting RSA was related to more frequent cannabis use. Individuals with larger declines in RSA from the video to the task (i.e., greater PNS responses) tended to use each substance more frequently. RSA recovery from the task was not related to substance use. Taken together, college students who are more physiologically responsive to challenge may use substances more frequently, potentially as a means of coping. Biofeedback interventions can be investigated for reducing college students' substance use risk.

## 1 Introduction

Nearly two‐thirds (63.5%) of college students reported using alcohol in the past 3 months, and nearly half (43.8%) reported using cannabis in their lifetime (American College Health Association, 2023). Frequent substance use can lead to social and academic consequences for college students (Patrick et al. 2020) and increased risk for substance use disorders (SUDs) during this period of neurobiological vulnerability (Prince, Read, and Colder 2019). Because few studies have examined the physiological correlates of substance use, the present study assessed whether college students' physiological responses to an ambiguous challenge—a common experience for college students—is related to alcohol and cannabis use.

### 1.1 Resting Respiratory Sinus Arrhythmia and Substance Use

The autonomic nervous system controls involuntary activity (e.g., metabolism) and has two branches: the sympathetic nervous system (SNS; ‘fight‐or‐flight’ system), which mobilises energy when a threat is present, and the parasympathetic nervous system (PNS; ‘rest or digest’ system), which conserves energy. The PNS is thought to chronically suppress the SNS, such that high levels of PNS at rest can promote health and conservation of bodily resources (Porges 2007). Higher PNS activity at rest (i.e., during a neutral or relaxed state, without demanding stimuli) has been related to greater activation of cortical regions including the prefrontal cortex (Thayer et al. 2012). The neurovisceral integration model therefore posits that PNS activity can promote cognitive and affective processes (Thayer and Lane 2009). PNS activity can be measured with respect to high‐frequency heart rate variability, often with respect to respiratory sinus arrhythmia (RSA), which can be assessed using spectral analysis of electrocardiogram data. Higher RSA has been considered a transdiagnostic indicator of psychopathology (Beauchaine 2015). Higher resting levels of PNS activity with respect to heart rate variability have been related to pronounced use of emotion regulatory processes (Geisler et al. 2010; Gillie, Vasey, and Thayer 2015), which are involved in mental health as well as substance use (e.g., Weiss et al. 2022).

RSA has also been suggested as a treatment‐outcome in SUDs (Eddie et al. 2014 2015) because promoting one's ability to regulate emotion may support individuals in SUD treatment. Individuals often report using substances to regulate emotion in line with stress‐coping and tension‐reduction models of substance use (Cappell and Greeley 1987; Conger 1956; Votaw and Witkiewitz 2021), and heightened emotion can increase odds of daily use (Dora et al. 2023a, 2023b). Biofeedback has reduced cravings for alcohol in adults with SUDs (Eddie et al. 2014), suggesting that difficulties with physiological reactivity to stimuli could contribute to substance use. It remains unclear whether resting RSA likewise relates to substance use in college students.

### 1.2 Respiratory Sinus Arrhythmia Responses and Substance Use

In addition to resting levels, changes in PNS activity in response to stimuli could suggest sensitivity to stress. Following stress, PNS activity tends to decline (i.e., vagal withdrawal), enabling an increase in sympathetic nervous system activity according to polyvagal theory (Porges 2007). Some individuals have larger physiological responses than others, which could suggest that they are experiencing greater cognitive‐emotional responses to the environment that tax psychobiological systems (Chida and Steptoe 2010; Pang and Beauchaine 2013).

RSA reactivity—one index of changes in PNS activity at the heart—has been related to mental health (Graziano and Derefinko 2013) and substance use (e.g., Eddie et al. 2014; Rahal et al. 2022), but associations have been mixed. One study of adolescents found that dampened parasympathetic and sympathetic nervous system responses to intensive social‐evaluative stress were associated with use of more substances two years later among non‐users (Rahal et al. 2022). Associations differed among youth who had used substances by age 14, who might be positioned for relatively riskier use, such that greater autonomic responses were related to use of more substances two years later.

Although past studies have assessed RSA responses to stress (e.g., Rahal et al. 2022), college students typically use substances beyond stressful contexts (Dora et al. 2023a), including to promote positive emotion and build friendships (Votaw and Witkiewitz 2021). Therefore, physiological responses to challenge (i.e., situations perceived to be stressful but manageable; Tomaka et al. 1997) generally—rather than solely threatening circumstances—may be consequential for substance use. College students regularly experience challenges (Costello, Copeland, and Angold 2011), particularly following the advent of the COVID‐19 pandemic and the transitions between online and in‐person education (Dave, Jaffe, and O'Shea 2024). Students differ in their ability to view stressors as positive challenges versus threats (Kilby, Sherman, and Wuthrich 2018), and negative appraisals such as viewing stress as debilitating are impactful and related more intensive alcohol use (Brenman et al. 2024). Individuals also show physiological responses to cognitive and academic tasks (Holzman and Bridgett 2017; Scrimin et al. 2019), which have implications for their substance use. For example, students who highly engage with academics in spite of challenges tend to abstain from substance use (e.g., Froiland et al. 2021). Although individuals differ in how they physiologically respond to daily challenges, few studies have assessed the implications of PNS responses to a challenge for substance use.

### 1.3 Present Study

The present study assessed whether college students' responses to an acute challenge—a novel, difficult mirror‐tracing task—would relate to frequency of alcohol and cannabis use. Despite high prevalence of substance use among college students (American College Health Association 2023), assessment of associations between RSA and substance use has been primarily limited to addiction treatment (Eddie et al. 2014). We addressed associations between substance use and, in line with prior studies (Rahal et al. 2022), both resting RSA and RSA responses as indicators of PNS activity and reactivity, respectively. We had two hypotheses. First, in line with evidence that higher resting RSA is posited to confer resources for affective regulation (Gillie, Vasey, and Thayer 2015; Porges 2007), we hypothesised that college students with lower resting RSA would use alcohol and cannabis more frequently. Additionally, greater physiological responses to stress could suggest greater vulnerability to stress and tax one's health (Lovallo and Gerin 2003). Therefore, our second hypothesis was that college students with greater PNS responses (i.e., greater withdrawal) to acute challenge would use alcohol and cannabis more frequently.

## 2 Method

### 2.1 Participants

College students (*N* = 152; *M*_age_ = 20.5, *SD* = 3.2; 73.8% female) from a public institution in Southern California were compensated with one hour of course credit. This sample size was selected based on norms from previous studies of acute physiological reactivity and a recent review indicating that a case‐control design assessing RSA would require a sample of 94 participants to detect a medium effect size (Quintana 2017). Students from all class years participated (19.2% first‐year, 18.5% second‐year, 28.5% third‐year, 33.8% fourth‐year). Participants had varied racial/ethnic backgrounds (28.5% white, 31.8% Asian, 15.9% Latine, 15.2% multiracial, 8.6% different identities including Black or Middle Eastern). Participants rated each parent's level of education, and values were averaged when possible (1 = less than high school, 2 = high school graduate, 3 = some college, 4 = 2‐year degree, 5 = 4‐year degree, 6 = professional degree, 7 = doctorate). Most participants' parents averaged between a 2‐year degree and a 4‐year degree (*M* = 4.58, *SD* = 1.71, range 1–7; 60% averaged a 4‐year college degree or higher education).

### 2.2 Procedure

Participants provided consent and then had electrocardiogram electrodes administered on the upper right chest below the clavicle, bottom of the lower right rib, and middle of the lower left rib in a lead II configuration. Participants reported past month substance use and then watched a 4‐min neutral video about the environment to establish a resting baseline. They then completed a mirror‐tracing task (i.e., challenge); they traced a star as quickly and closely as possible with their nondominant hand while only seeing the mirror reflection due to a board blocking the paper (e.g Hinnant et al. 2016, 2022). This well‐established problem‐solving task tends to elicit PNS responses in children (Matthews, Woodall, and Stoney 1990), which have been related to children's substance use (Hinnant et al. 2016 2022). This task was used because it was anticipated to be less stressful for adults than for children and therefore provide an ambiguous challenge for adults. As a validity check, participants reported whether they viewed the task as a positive challenge and as stressful, frustrating, and threatening on a 7‐point scale. On average participants considered the task a positive challenge (*M* = 5.21, *SD* = 1.26, range 1–7), and a one‐sample *t*‐test indicated that ratings exceeded the scale midpoint (*t* [151] = 11.79, *p* < 0.001). When rating whether they felt challenged on a six‐point scale, participants generally rated above a 4 (*agree to some extent*; *M* = 4.73, *SD* = 1.08; *t* [151] = 8.31, *p* < 0.001). Participants generally did not find the task stressful (*M* = 3.24, *SD* = 1.82), frustrating (*M* = 3.75, *SD* = 1.77), or threatening (*M* = 1.46, *SD* = 0.90), with ratings of frustration near the midpoint (*t* [151] = 1.74, *p* = 0.084) and ratings of the task as stressful (*t* [151] = 5.12, *p* < 0.001) and threatening (*t* [151] = 34.73, *p* < 0.001) significantly below the midpoint.

After completing the challenge task, participants could choose to reattempt and have their best performance evaluated. Participants had between one and three attempts (32.5% one attempt, 38.4% two attempts, 29.1% three attempts). This approach allowed for participants to engage with the task for a longer time and for participants to discontinue if they experienced fatigue. Participants then reported attitudes about the task in a post‐task survey. All procedures were approved by the University of California, Los Angeles Institutional Review Board. Participants completed informed consent, which affirmed anonymity and that they could discontinue the study at any point and still receive course credit. The Principal Investigator was not teaching any undergraduate classes, reducing the likelihood that students would feel required to participate. All study data were deidentified and stored in Qualtrics.

### 2.3 Measures

#### 2.3.1 Substance Use Frequency

Participants completed separate items for alcohol and cannabis (including vaping) use. Participants reported how often each substance was used over the last 30 days using a 7‐point scale (1 = 0 days, 2 = 1–2 days, 3 = 3–5 days, 4 = 6–9 days, 5 = 10–19 days, 6 = 20–29 days, 7 = all 30 days). For alcohol, they were additionally asked about the frequency of binge drinking, defined as 4+ drinks within a couple of hours for females and 5+ drinks within a couple of hours for males, on a 7‐point scale (1 = 0 days, 2 = 1 day, 3 = 2 days, 4 = 3–5 days, 5 = 6–9 days, 6 = 10–19 days, 7 = 20+ days).

#### 2.3.2 Respiratory Sinus Arrhythmia

Electrocardiogram data were collected continuously during the nature video, all attempts of the task, and the post‐task survey using a BIOPAC physiological recording system. Data were converted to inter‐beat intervals, and artefacts were edited by three research assistants certified CardioEdit Reliable (Brain‐Body Center 2007). All files were double edited, with near identical values across research assistants (*r* = 0.97, *p* < 0.001). RSA was estimated from the software CardioBatch across high frequencies (0.12–0.40 Hz) using the Porges–Bohrer Method over 30‐s epochs, natural log‐transformed to produce normal distributions, and then averaged across epochs (Porges 2007; Porges and Bohrer 1990; Riniolo and Porges 2000).

### 2.4 Analytic Plan

We used piecewise multilevel models with task segments nested within participants to examine changes in RSA during the 4‐min video, the star‐tracing task (*M* = 5.96, *SD* = 3.81), and the post‐task survey. To have time intervals with comparable length, only the first 5‐min period of the post‐task survey was used for analysis, and 5‐min periods are common for RSA assessment (Camm et al. 1996). Segments were dummy‐coded to characterise both reactivity (i.e., change from during the video to during the task; video = −1, star‐tracing task and post‐task survey = 0 for reactivity) and recovery (i.e., change from the task to the post‐task survey; video and star‐tracing task = 0, post‐task survey = 1 for recovery; Hastings and Kahle 2019). Dummy codes were included as random slopes, such that the magnitude of change could randomly vary across participants. This approach was used because multilevel models allow for all available data to be leveraged, account for nesting of values within individuals, and simultaneously analyse reactivity and recovery while accounting for baseline RSA (Hastings and Kahle 2019; Lehman, Kirsch, and Jones 2015). These models require that the lowest level of observation (i.e., RSA across task segments) is specified as the outcome, with higher level variables (i.e., person‐level variables including substance use) as predictors. Estimates from these models enable visualisation of the PNS response across the task and leverage all data per participant.

After characterising the overall PNS response, we tested whether PNS responses differed by participants' substance use by testing Task Segment × Substance Use interactions, with separate models per substance. Effect sizes were estimated as the percentage of the random slope accounted for by including the cross‐level interaction (Aguinis, Gottfredson, and Culpepper 2013). Simple slopes were probed for significant interactions by examining the PNS response at different levels of substance use (i.e., mean and one standard deviation above and below the mean). Models controlled for age, race (dummy‐coded relative to Asian, sample majority), sex (dummy‐coded relative to female, sample majority), sexual orientation (dummy‐coded relative to heterosexual, sample majority), and parents' education because these demographic factors are consistently related to substance use (Gardner et al. 2020; Martin 2019; Schipani‐McLaughlin et al. 2022). Unadjusted models resulted in an identical pattern of results. Multilevel models account for missing data using maximum likelihood and assume normality of the outcome (RSA skewness = −0.10, kurtosis = 2.65), not predictor variables.

## 3 Results

Most participants reported drinking alcohol in the past 30 days (72.2%), and over half reported binge drinking (55.0%). One third of participants reported using cannabis in the past 30 days (37.1%). On average, participants reported drinking alcohol on between 1 and 5 days (*M* = 2.55, *SD =* 1.33), binge drinking alcohol on one day (*M* = 2.26, *SD =* 1.48), and using cannabis on 1–2 days of the past 30 days (*M* = 2.01, *SD =* 1.66). The full range of cannabis use was endorsed, including daily use. Frequency of alcohol use, binge drinking, and cannabis use were positively correlated (Table 1). Higher resting RSA was correlated with more frequent cannabis use, whereas greater RSA reactivity was related to more frequent alcohol use, binge drinking, and cannabis use.

**TABLE 1:** Descriptive statistics and correlations between study variables.

Variable	*M*	*SD*	1.	2.	3.	4.	5.	6.	7.	8.	9.	10.
1. Baseline RSA	6.53	1.21	—									
2. RSA reactivity	0.40	0.75	0.48***	—								
3. RSA recovery	−0.25	0.61	−0.23**	−0.66***	—							
4. Alcohol frequency	2.54	1.33	0.11	0.16*	−0.07	—						
5. Binge frequency	2.26	1.48	0.14	0.22**	−0.10	0.82***	—					
6. Cannabis frequency	1.98	1.63	0.21***	0.20*	−0.09	0.25***	0.26***	—				
7. Sex	0.26	0.44	0.08	0.04	0.05	0.00	−0.03	0.10	—			
8. Sexual orientation	0.23	0.42	−0.04	0.02	0.06	−0.02	0.04	0.14	−0.04	—		
9. Parents' education	4.57	1.71	0.15	0.13	−0.01	0.04	0.20*	−0.04	0.11	0.01	—	
10. Age	20.08	1.26	0.04	0.07	0.05	0.20*	0.07	0.11	0.13	0.03	−0.18*	—

Abbrevations: RSA = Respiratory sinus arrythmia. RSA Reactivity = Baseline RSA—Task RSA. RSA Recovery = Recovery RSA—Baseline RSA.

*p < 0.05, **p < 0.01, ***p < 0.001.

Regression models tested whether resting RSA was related to substance use. Resting RSA was not significantly related to either alcohol use or binge drinking, *p*s > 0.17 (Table 2). In contrast to the hypothesis, higher resting RSA was related to more frequent cannabis use (*B* = 0.28, *SE* = 0.11, *β* = 0.21, 95% Confidence Interval [CI] [0.07, 0.51], *p* = 0.009).

**TABLE 2:** Substance use as a function of baseline parasympathetic nervous system activity using linear regression.

	Alcohol Frequency		Binge Frequency		Cannabis Frequency	
	*B*	*SE*	*B*	*SE*	*B*	*SE*
Intercept	2.12***	0.26	1.99***	0.29	1.41***	0.34
Baseline RSA	0.10	0.09	0.14	0.10	0.28*	0.11
Sex	−0.15	0.24	−0.26	0.27	0.30	0.32
Sexual orientation	−0.10	0.25	0.12	0.28	0.56	0.32
Race						
Latine	−0.52	0.37	−0.52	0.42	0.40	0.48
White	0.61*	0.27	0.53	0.30	0.50	0.35
Multiracial	0.14	0.32	0.20	0.36	0.06	0.42
Different identity	−0.67	0.40	−0.51	0.45	0.47	0.53
Parents' education	−0.03	0.08	0.09	0.09	−0.06	0.10
Age	0.21*	0.09	0.13	0.10	0.06	0.11

*Note:* Baseline RSA and Parents' Education were centred at the sample mean. Race was dummy‐coded relative to Asian, sample majority. Sex was dummy‐coded relative to female, sample majority. Sexual orientation was dummy‐coded relative to heterosexual, sample majority. Age was centred at 18, and values above 22 were recoded as 22.

Abbrevation: RSA = Respiratory sinus arrhythmia.

*p < 0.05, **p < 0.01, ***p < 0.001.

Models tested PNS activity across the task (Supporting Information S1: Table S1). On average, participants showed vagal withdrawal (i.e., decreased RSA) from baseline to during the challenge task (*B* = 0.39, *SE* = 0.05, 95% CI [0.29, 0.50], *p* < 0.001). They also recovered, with an increase in RSA from the challenge task to the post‐task survey (*B* = 0.25, *SE* = 0.05, 95% CI [0.15, 0.34], *p* < 0.001). Differences in PNS responses emerged by race/ethnicity, but not by age, sex, sexual orientation, or parents' education (all *p*s > 0.07; Supplemental Information). No differences by demographic factors emerged in associations between substance use and RSA.

**FIGURE 1:**
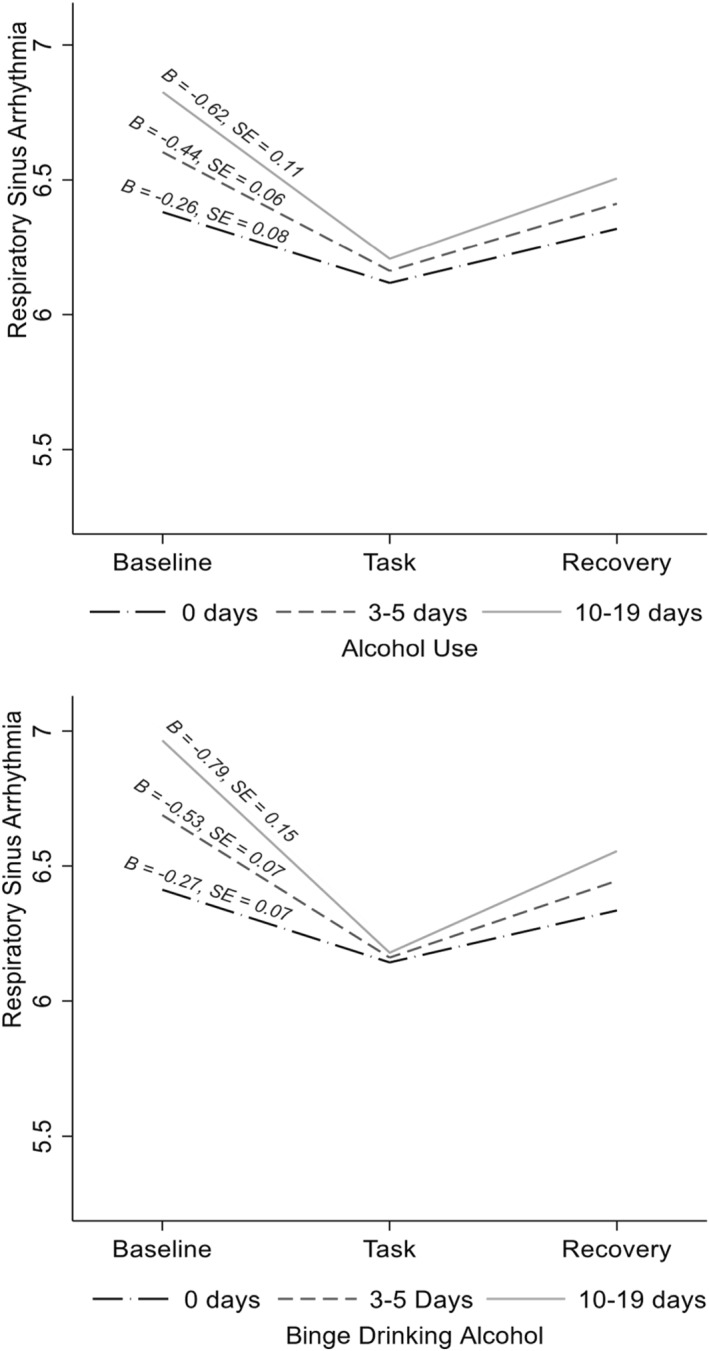
Respiratory sinus arrythmia responses to the task as a function of alcohol use (above) and binge drinking (below). Simple slopes were probed at different levels of past month frequency of use. Differences from baseline to the task are all significant, *p*s < 0.001, although the degree of reactivity significantly differed by frequency of alcohol use (*B* = 0.09, *SE* = 0.04, *p* = 0.012) and binge drinking (*B* = 0.12, *SE* = 0.04, *p* = 0.001). Analyses controlled for age, race (dummy‐coded relative to Asian, sample majority), sex (dummy‐coded relative to female, sample majority), sexual orientation (dummy‐coded relative to heterosexual, sample majority), parents' education, and age.

**FIGURE 2:**
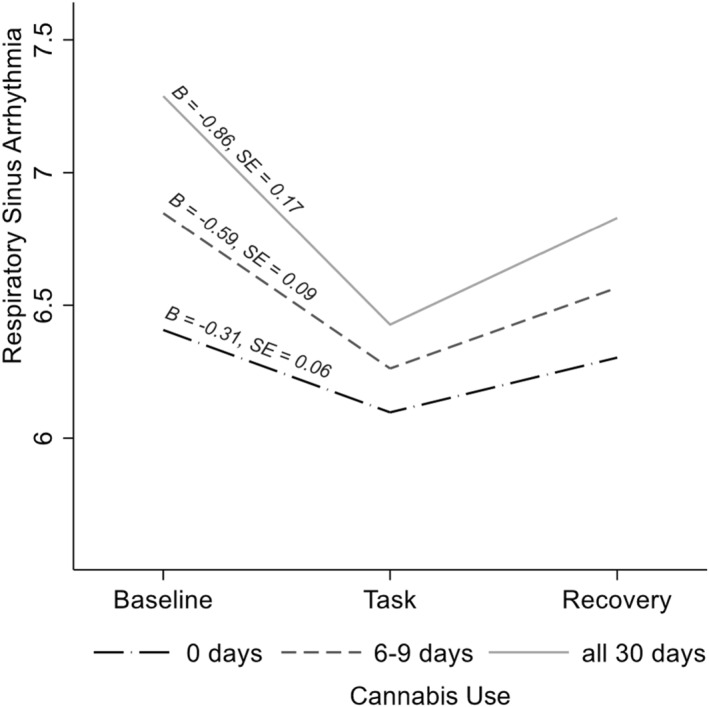
Respiratory sinus arrythmia responses to the task as a function of cannabis use. Simple slopes were probed at different levels of past month frequency of cannabis use. Differences from baseline to the task are all significant, *p*s < 0.001, although the degree of reactivity significantly differed by frequency of cannabis use (*B* = 0.09, *SE* = 0.03, *p* = 0.009). Analyses controlled for age, race (dummy‐coded relative to Asian, sample majority), sex (dummy‐coded relative to female, sample majority), sexual orientation (dummy‐coded relative to heterosexual, sample majority), parents' education, and age.

As hypothesised, more frequent past month alcohol use (*B =* 0.09, *SE =* 0.04, 95% CI [0.02, 0.18], *p* = 0.012, 13.4% random slope variance of reactivity slope), binge drinking (*B =* 0.12, *SE =* 0.04, 95% CI [0.05, 0.19], *p =* 0.001, 18.1% random slope variance of reactivity slope), and cannabis use (*B =* 0.09, *SE =* 0.03, 95% CI [0.02, 0.15], *p =* 0.009, 17.0% random slope variance of reactivity slope) were related to greater PNS responses to the task (Figures 1, 2; Supporting Information S1: Table S2). Simple slopes analysis indicated that most individuals showed PNS reactivity to the task (i.e., vagal withdrawal), but individuals who used each substance more frequently had a larger degree of PNS reactivity. Recovery did not differ by alcohol use, binge drinking, or cannabis use (*p*s > 0.13).

Finally, supplemental models tested reactivity and recovery, calculated as difference scores (i.e., Task—Nature Video; Post‐Task Survey—Task, respectively), as predictors of substance use in a linear regression framework. In alignment with results from multilevel models, regression models indicated that greater RSA reactivity was related to marginally more frequent alcohol use (*B =* 0.32, *SE =* 0.19, *β* = 0.18, 95% CI [−0.06, 0.71], *p =* 0.097) and significantly more frequent binge drinking (*B =* 0.48, *SE =* 0.22, *β* = 0.24, 95% CI [0.04, 0.93], *p =* 0.032) and cannabis use (*B =* 0.54, *SE =* 0.25, *β* = 0.25, 95% CI [0.04, 1.03], *p =* 0.033), with no associations emerging for recovery, *p*s > 0.6 (Supporting Information S1: Table S3).

## 4 Discussion

The present study aimed to characterise associations between college students' physiological responses to challenge and substance use frequency. Contrary to hypotheses, higher resting RSA was related to more frequent cannabis use, but not alcohol use or binge drinking. In turn, participants with greater RSA responses to challenge also tended to use alcohol, binge drink, and use cannabis more frequently. These results may suggest that how individuals physiologically respond to acute challenge is related to their frequency of substance use in ways distinct from their resting RSA activity. Individuals with a physiological predisposition for responding to acute challenge may also be more inclined to engage in risky substance use behaviour to either address arousal or cope with stress.

Higher resting RSA was hypothesised to relate to less frequent substance use because individuals with higher resting RSA tend to feel more socially connected and have greater emotion regulatory capacity (Kok and Fredrickson 2010; Wang, Lü, and Qin 2013). Therefore, associations between higher resting RSA and more frequent cannabis use were unexpected. Individuals are more likely to report coping motives for cannabis use than for alcohol use (Votaw and Witkiewitz 2021), which may explain why associations emerged for cannabis but not alcohol use. Although resting RSA and RSA reactivity are often related, prior studies have found associations between substance use and RSA reactivity but not resting RSA (Rahal et al. 2022). Higher resting RSA could suggest greater sensitivity to environmental stimuli (Porges 2007), including greater emotional responses to stress (Rahal et al. 2023). College is overwhelming for many students (Costello, Copeland, and Angold 2011), and higher resting RSA in stressful circumstances could relate to greater cannabis use as a means of coping. Moreover, cannabis acutely reduces PNS activity for occasional female users (Pabon et al. 2022) and may also thereby alter PNS responses over time.

Individuals with greater RSA responses to the challenge task also tended to use more frequently over the past month. This finding is at odds with past evidence that attenuated autonomic responses to acute stress prospectively predict the number of substances that adolescents used two years later (Rahal et al. 2022). However, rates of substance use and pathways to use change with age (Mayes and Suchman 2015), such that adolescents are at lower risk for frequent and intensive substance use than college students (American College Health Association 2023). Further, that study examined how RSA responses to acutely distressing social‐evaluative stress relate to whether adolescents had ever used a substance, and the implications of reactivity are thought to differ between severe stress versus moderate stress, which may be similar to challenge (Vrshek‐Schallhorn et al. 2018). Individuals with dampened RSA responses to stress may be unable to mount a physiological response to intensive stress and therefore use substances to elicit physiological arousal (Chaplin, Niehaus, and Gonçalves 2018). In turn, greater RSA responses to ambiguous challenge stimuli could suggest greater environmental sensitivity and thereby greater risk for using substances for either coping or social reasons, such as the approval of others.

Rather than RSA responses conferring risk for substance use, substance use could also impact RSA responses by disrupting how sensitive individuals are to stimuli. Frequent substance use could disrupt one's motivation to continue to engage with challenge tasks, such that individuals need greater physiological resources to respond to the task. There is ample evidence that repeated use can dysregulate psychobiological systems among individuals with SUDs (Eddie et al. 2021; Lovallo 2006), but such effects are less common among less frequent users.

Substance use was related to reactivity to challenge but not recovery, or one's ability to return to baseline following a stimulus. It is possible that the physiological arousal related to a larger acute RSA response could contribute to cravings (Eddie et al. 2014) or motivate substance use as a means of relaxing or reducing arousal (Chaplin, Niehaus, and Gonçalves 2018). Recovery from challenge is sometimes more related to health than reactivity, as an inability to recover can result in long‐term physiological arousal (Almeida et al. 2020). However, recovery was measured by participants' physiological activity during a survey asking them to reflect on their performance. Such thoughts could elicit physiological activation, contaminating estimates of recovery. It is possible that associations with recovery may have emerged if participants continued the baseline video or were distracted from the task.

### 4.1 Implications

Study findings suggest that RSA responses are related to substance use behaviours beyond clinical populations (Eddie et al. 2015). Physiological responses could be one means of identifying at‐risk individuals for educational and skill‐based trainings (Larimer and Cronce 2002). Promoting awareness of bodily sensations including physiological responses can promote self‐regulation and reduce long‐term stress (Schultchen et al. 2019), which may impact substance use. Greater psychophysiological responses to acute challenge could suggest difficulty coping with challenge, and college students encounter academic, social, and autonomy‐related challenges. Interventions that target how students view challenge (e.g., growth mindset or a stress‐is‐enhancing mindset) may have downstream effects on physiological responses and ultimately substance use (e.g., Crum et al. 2017). Instructors and adult role models can emphasise the ubiquitousness of challenge, positively frame challenge, and foster environments that allow students to embrace challenge. Because findings were cross‐sectional, substance use could theoretically impact how students physiologically respond to acute challenge. Mobilisation of psychobiological systems can impact learning and memory and could be one mechanism by which substance use relates to poorer academic performance, particularly for frequent users.

### 4.2 Limitations

The study was cross‐sectional, precluding directional conclusions. Prospective studies are needed to examine bidirectional associations between physiological responses and substance use. The study was limited to college students, who tend to engage in substance use in different contexts from young adults who do not attend college (Patrick et al. 2020). Findings are also likely unique to the challenge used in this study, which has previously been used in youth. Future studies using this task in adults, as well as other challenge tasks, are needed to better contextualise results. Finally, substance use measures were limited by the lack of contextual factors (e.g., motives, solitary use) and the omission of illicit substances.

## 5 Conclusions

When examining the association between college students' PNS responses to ambiguous challenge and substance use, we found that more frequent past month substance use was related to greater PNS reactivity to a challenge task, but not recovery from the task, suggesting that difficulty responding to challenge is related to substance use. These findings extend past research by testing associations between PNS reactivity to challenge and substance use in college students, a group with high substance use risk (Costello, Copeland, and Angold 2011). Findings suggest that psychoeducation regarding awareness of bodily responses (Schultchen et al. 2019), interventions mindsets regarding challenge versus stress may impact substance use (Crum et al. 2017), and physiological biofeedback interventions (Eddie et al. 2014) should be examined as means of reducing substance use risk in college students.

## Consent

The study was approved by the University of California, Los Angeles. All participants provided informed consent.

## Conflicts of Interest

The authors declare no conflicts of interest.

## Supporting information

Supporting Information S1

## Data Availability

The data that support the findings of this study are available from the corresponding author upon reasonable request. The data necessary to reproduce the analyses presented here are not publicly accessible. The analyses presented here were not preregistered.
