# Supplementary material for: Substance Use is Associated With College Students' Acute Parasympathetic Nervous System Responses to Challenge
Source: Stress Health. 2025 Jan 20;41(1):e70002. doi: 10.1002/smi.70002 (PMC11745212; doi:10.1002/smi.70002)
Supplement: Supplementary file 1 — Supporting Information S1 [file SMI-41-e70002-s001.pdf]

Supplemental Information for Substance Use is Associated with College Students' Acute Parasympathetic Nervous System Responses to Challenge

Table S1 presents parasympathetic nervous system response to the task across the sample in a multilevel framework. Results regarding demographic differences in the parasympathetic nervous system response to the task are then summarized. Table S2 presents parasympathetic nervous system response to the task as a function of substance use in a multilevel framework. Table S3 presents substance use as a function of parasympathetic nervous system response to the task. Stata 16.1 syntax for all analyses and measures for substance use frequency and demographic information are then provided.

Table S1. Parasympathetic nervous system activity as a function of task.

	Outcome: Respiratory Sinus Arrythmia			
	B	*SE*	[95% Conf. Interval]	
Intercept	5.90***	0.21	5.48	6.31
Difference from Baseline	0.41***	0.06	0.30	0.52
Difference from Recovery	0.25***	0.05	0.15	0.34
Sex	0.09	0.20	-0.30	0.47
Sexual Orientation	-0.11	0.20	-0.50	0.28
Race				
Latine	0.08	0.30	-0.51	0.67
White	-0.07	0.22	-0.50	0.36
Different Identity	0.13	0.26	-0.39	0.64
Multiracial	0.60	0.32	-0.04	1.23
Parents' Education	0.08	0.06	-0.04	0.20
Age	0.08	0.07	-0.05	0.22

Note: *p<.05, **p<.01, ***p<.001. Sex was dummy-coded relative to female, sample majority. Sexual orientation was dummy-coded relative to heterosexual, sample majority. Race was dummy-coded relative to Asian, sample majority. Parents' Education was centered at the sample mean. Age was centered at 18, and values above 22 were recoded as 22.

Differences by demographic factors were tested by incorporating Segment × Demographic interactions. No differences emerged by sex, sexual orientation, parents' education, or age (all *p*s > .07). Differences in reactivity emerged by race, such that white participants had a significantly larger degree of vagal withdrawal relative to Asian (*B* = -0.41, *SE* = 0.14, 95% CI [-0.68, -0.13], *p* = .004) and individuals from different identities (*B* = -0.43, *SE* = 0.21, 95% CI [-0.84, -0.04], *p* = .040), with marginal differences emerging in Latine students (*B* = -0.33, *SE* = 0.17, 95% CI [-0.66, 0.001], *p* = .051). Probing of simple slopes indicated that a significant degree of reactivity in vagal withdrawal emerged for individuals from all racial and ethnic backgrounds except for those from different racial identities (*B* = 0.16, *SE* = 0.18, 95% CI [-0.20, 0.52], *p* = .394). No differences by race emerged in recovery (all *p*s > .2).

Table S2. Parasympathetic nervous system activity as a function of task and substance use.

	Outcome: Respiratory Sinus Arrythmia					
	As a Function of Alcohol Use		*As a Function of Binge Drinking*		*As a Function of Cannabis Use*	
	B	*SE*	*B*	*SE*	*B*	*SE*
Intercept	5.92***	0.21	5.91***	0.21	5.98***	0.21
Difference from Baseline	0.41***	0.05	0.41***	0.05	0.41***	0.05
Difference from Recovery	0.25***	0.05	0.25***	0.05	0.25***	0.05
Substance Use	0.01	0.07	-0.01	0.06	0.07	0.05
Difference from Baseline × Substance Use	0.09*	0.04	0.12***	0.04	0.09**	0.03
Difference from Recovery × Substance Use	0.03	0.04	0.04	0.03	0.02	0.03
Sex	0.09	0.20	0.10	0.20	0.05	0.20
Sexual Orientation	-0.10	0.20	-0.11	0.20	-0.17	0.20
Race						
Latine	0.11	0.30	0.10	0.30	0.04	0.30
White	-0.09	0.22	-0.08	0.22	-0.13	0.22
Multiracial	0.12	0.26	0.12	0.26	0.11	0.26
Different Identity	0.62	0.33	0.61	0.33	0.53	0.32
Parents' Education	0.08	0.06	0.08	0.06	0.08	0.06
Age	0.07	0.07	0.08	0.07	0.07	0.07

Note: *$p<.05$, **$p<.01$, ***$p<.001$. Sex was dummy-coded relative to female, sample majority. Sexual orientation was dummy-coded relative to heterosexual, sample majority. Race was dummy-coded relative to Asian, sample majority. Parents' Education were centered at the sample mean. Age was centered at 18, and values above 22 were recoded as 22.

Table S3. Substance use as a function of parasympathetic nervous system responses to the task using linear regression.

	Outcome: Frequency of Alcohol Use		*Outcome: Frequency of Binge Drinking*		*Outcome: Frequency of Cannabis Use*	
	B	*SE*	*B*	*SE*	*B*	*SE*
Intercept	2.07***	0.27	1.87***	0.31	1.26***	0.35
RSA Reactivity	$0.32^{\pm}$	0.19	0.48*	0.22	0.54*	0.25
RSA Recovery	0.10	0.23	0.13	0.27	0.16	0.30
Sex	0.04	0.25	-0.11	0.29	0.19	0.32
Sexual Orientation	-0.14	0.24	0.10	0.28	0.61	0.32
Race						
Latine	-0.72	0.37	-0.59	0.43	0.43	0.48
White	0.50	0.28	0.36	0.32	0.30	0.36
Multiracial	0.04	0.32	0.09	0.37	0.04	0.41
Different Identity	-0.74	0.40	-0.55	0.46	0.65	0.52
Parents' Education	-0.02	0.08	0.09	0.09	-0.07	0.10
Age	0.21	0.09	0.13	0.10	0.07	0.11

Note: $^{\pm}p<.1$, $*p<.05$, $***p<.001$. RSA = Respiratory sinus arrhythmia. RSA Reactivity (i.e., Task RSA – Baseline RSA), RSA Recovery (i.e., Post-Task RSA – Baseline RSA), and Parents' Education were centered at the sample mean. Sex was dummy-coded relative to female, sample majority. Sexual orientation was dummy-coded relative to heterosexual, sample majority. Race was dummy-coded relative to Asian, sample majority. Age was centered at 18, and values above 22 were recoded as 22.

Stata Syntax

```
*Associations between Alcohol Frequency, Binge Drinking Frequency, and Baseline RSA
regress AlcFreq RSA_Baseline i.Sex i.SexO ib1.Race ParEdu Age if Video==1, b
regress BingeFreq RSA_Baseline i.Sex i.SexO ib1.Race ParEdu Age if Video==1, b
regress CanFreq RSA_Baseline i.Sex i.SexO ib1.Race ParEdu Age if Video==1, b

*Associations between Alcohol Frequency, Binge Drinking Frequency, and RSA Responses in a Regression Framework
regress AlcFreq RSAReact RSARecov i.Sex i.SexO ib1.Race ParEdu Age if Video==1, b
regress BingeFreq RSAReact RSARecov i.Sex i.SexO ib1.Race ParEdu Age if Video==1, b
regress CanFreq RSAReact RSARecov i.Sex i.SexO ib1.Race ParEdu Age if Video==1, b

*Associations between Alcohol Frequency, Binge Drinking Frequency, and RSA Responses in a Multilevel Framework
mixed RSA c.AlcFreq_mc##ib1.VideoRecoveryContrast i.SexO ib1.Race ParEdu Age || ID: ib1.VideoRecoveryContrast, var cov()
mixed RSA c.BingeFreq_mc##ib1.VideoRecoveryContrast i.SexO ib1.Race ParEdu Age || ID: ib1.VideoRecoveryContrast, var cov()
mixed RSA c.PotFreq_mc##ib1.VideoRecoveryContrast i.SexO ib1.Race ParEdu Age || ID: ib1.VideoRecoveryContrast, var cov()
```

Substance Use Measures

Have you ever drank alcohol (more than a few sips)? If so, at what age did you have your first drink of alcohol (more than a few sips)?

- ○ [ ] Yes
- ○ No

Have you ever used marijuana (including vaping)? If so, at what age did you first use marijuana?

- ○ [ ] Yes
- ○ No

How often did you drink during the past month?

- ○ 0 days
- ○ 1-2 days
- ○ 3-5 days
- ○ 6-9 days
- ○ 10-19 days
- ○ 20-29 days
- ○ all 30 days

During the past 30 days, on how many days did you have **4** or more drinks of alcohol in a row, that is, within a couple of hours (if you are **female**) or **5** or more drinks of alcohol in a row, that is, within a couple of hours (if you are **male**)?

- ○ 0 days
- ○ 1 day
- ○ 2 days
- ○ 3-5 days
- ○ 6-9 days
- ○ 10-19 days
- ○ 20+ days

How often did you use marijuana over the past month (including vaping marijuana)?

- ◯ 0 days
- ◯ 1-2 days
- ◯ 3-5 days
- ◯ 6-9 days
- ◯ 10-19 days
- ◯ 20-29 days
- ◯ all 30 days

Demographics

How old are you?

What year in college are you?

- ◯ First
- ◯ Second
- ◯ Third
- ◯ Fourth
- ◯ More than fourth

What is your gender?

What is your sexual orientation?

My ethnicity is

- ◯ South Asian
- ◯ East Asian
- ◯ South East Asian
- ◯ Other Asian Ethnicity
- ◯ Black or African American
- ◯ Hispanic or Latino, including Mexican American, Central American, and others
- ◯ White, Caucasian, Anglo, European American; not Hispanic
- ◯ American Indian/Native American
- ◯ Arab or Arab American
- ◯ Persian
- ◯ Mixed; Parents are from two different groups (please specify) ____
- ◯ ____ Other (please specify)

What is your mother's education?

- ◯ Less than high school
- ◯ High school graduate
- ◯ Some college
- ◯ 2 year degree
- ◯ 4 year degree
- ◯ Professional degree
- ◯ Doctorate
- ◯ Don't Know

What is your father's education?

- ◯ Less than high school
- ◯ High school graduate
- ◯ Some college
- ◯ 2 year degree
- ◯ 4 year degree
- ◯ Professional degree
- ◯ Doctorate
- ◯ Don't Know
